# Metabolomic profiling in a rat model of visual fatigue associated with liver-kidney yin deficiency syndrome

**DOI:** 10.3389/fendo.2025.1586581

**Published:** 2025-07-28

**Authors:** Xue Zhang, Xin Liu, Zhiqiang Huang, Yan Chen, Yingli Zhu, Jianjun Zhang, Jun Du, Liang Chen

**Affiliations:** ^1^ Amway Phytonutrients Research Center, Nutrilite (China) Nutrition & Health Institute, Shanghai, China; ^2^ Department of Ophthalmology, Union Hospital, Tongji Medical College, Huazhong University of Science and Technology, Wuhan, China; ^3^ School of Chinese Materia Medica, Beijing University of Chinese Medicine, Beijing, China; ^4^ School of Traditional Chinese Medicine, Beijing University of Chinese Medicine, Beijing, China

**Keywords:** visual fatigue, liver and kidney yin deficiency, UHPLC-Q-Exactive Orbitrap/MS, metabolomics, signaling pathways

## Abstract

**Background:**

Visual fatigue, commonly attributed to excessive eye use or dry conditions, is traditionally associated with deficiencies in liver and kidney yin in Chinese medicine. However, its metabolic aspects remain largely unexplored.

**Methods:**

Levothyroxine sodium combined with tail-clip stimulation induced a rat model of visual fatigue with liver and kidney yin deficiency. At 3 (M1), 7 (M2) and 14 (M3) days after induction, histopathological changes were observed, and metabolic profiling was completed using untargeted UHPLC-Q-Exactive Orbitrap/MS.

**Results:**

The rats exhibited signs of liver and kidney yin deficiency and visual fatigue on days 3 and 7, respectively. Compared to the control group, we identified 127 and 96 differential metabolites in the serum on days 7 and 14, respectively, primarily lipids and organic nitrogen compounds. Moreover, we observed disruptions in sphingolipid metabolism and signaling pathways.

**Conclusion:**

This study enriches our understanding of the metabolic profile associated with liver-kidney-yin deficiency type visual fatigue.

## 1 Introduction

Visual fatigue is caused by a variety of factors, including excessive eye use and environmental or pathological factors, which are mainly manifested as frequent eye discomfort, such as ocular acid, dryness, and blurred vision (1, 2). It is common in both children and adults. Statistically, the prevalence of visual fatigue in children (< 18 years old) ranges from 12.4–32.2% (3, 4), and that in college students worldwide is 46–71% (5). In addition, the number of people suffering from visual fatigue is increasing annually. These people come from all age groups, especially those who frequently use digital devices such as computers (6). The dangers of visual fatigue are obvious, which not only affects the state of study and work but may also cause the progression of diseases such as dry eye and video terminal syndrome (7). Therefore, considering the impact of visual fatigue on physical and mental health, the identification of methods to alleviate visual fatigue is necessitated.

To improve clinical prevention and treatment strategies for visual fatigue, we first need to understand the exact mechanism underlying visual fatigue. According to traditional Chinese medicine (TCM) theory, visual fatigue is related to the liver, kidneys, and lungs (8). Its etiology involves multiple aspects such as liver-kidney yin deficiency, qi blood deficiency, and stomach weakness (9). Zeng et al. classified visual fatigue into three types according to the dialectics of TCM–heart-blood deficiency, liver-kidney deficiency, and qi-blood deficiency, of which liver-kidney yin deficiency is the most common (10). Liver and kidney yin deficiency may cause eye discomfort such as dry eyes, tired eyes, and blurry vision (11). However, the etiology and biochemical basis of visual fatigue with liver-kidney yin deficiency remain unknown. Understanding the effects of liver and kidney yin deficiency on visual fatigue is expected to provide valuable information for clinical applications.

In traditional and modern medicine, metabolomics is an important technology for studying the networks of biological systems. Metabolomics describes the metabolic profiles of endogenous substances and their dynamic changes to characterize different physiological and pathological states of an organism (12). Recent studies have emphasized the application of metabolomics in understanding the pathophysiological mechanisms of liver diseases (13, 14) and kidney diseases (15, 16), advancing precision medicine and patient care. However, traditional research methods often fail to effectively clarify the potential of TCM therapeutic interventions, hindering the modern exploration of TCM-derived functional compounds. Metabolomics can accurately capture the molecular interactions among disease-responsive metabolites, thereby characterizing the systemic fluctuations and molecular complexity of TCM-derived functional compounds (17, 18). Clinical examinations have revealed that individuals with symptoms of liver and kidney yin deficiency exhibit enhanced energy metabolism and disturbed free radical metabolism (19). Moreover, micronutrients affect the morphology and function of eye tissues by participating in multiple cellular metabolic pathways to maintain the body’s homeostatic balance (20). Therefore, the identification of metabolite changes during the progression of visual fatigue can provide useful information for improving eye health. However, the metabolites and metabolic pathways involved in visual fatigue have not yet been identified.

High-resolution mass spectrometry (MS), especially UHPLC coupled with a Q-Exactive orbitrap/MS, is widely used to analyze the chemical composition of biological samples and herbal plants (21). The advantages of this technique include fast polarity switching and high sensitivity. Therefore, in this study, we utilized levothyroxine sodium combined with a tail clamp and light/dark environment manipulation to create a rat model of visual fatigue with liver and kidney yin deficiency. Then, through untargeted UHPLC-Q-Exactive orbitrap/MS metabolomics analysis identified differential metabolites and perturbed pathways closely related to visual fatigue progression, especially in sphingolipid metabolism and sphingolipid signaling pathway. The obtained data can help to better understand the metabolic characteristics associated with visual fatigue and, reveal the link between sphingolipid metabolic disorders and visual fatigue in the context of liver and kidney yin deficiency. This offers a new perspective for modern neuro-ophthalmology, metabolically corroborating the TCM theory that “liver and kidney yin deficiency leads to inadequate eye nutrition” (11, 22), shows the complementarity of TCM and Western medicine, thereby providing a new scientific basis for the targeted treatment of this disease.

## 2 Methods

### 2.1 Animal preparation

Sprague-Dawley (SD) male rats (specific pathogen-free (SPF) grade, license number SCXK (Jing) 2021–0011), weighting 200 ± 20 g, 6–7 weeks old, were acquired from Beijing Vital River Laboratory Animal Technology Co., Ltd. (Beijing, China) and housed in an SPF laboratory (license number SYXK (Shan) 2018–0009). Before performing the experiments, all animals were acclimatized under controlled conditions (20–25°C, 45–65% relative humidity, 12-h dark/light cycle) for five days, with free access to water and food. The research protocol was in accordance with the ethical requirements and authorized by the Animal Ethics Committee (ethics approval number 20220913). All methods were carried out in accordance with relevant guidelines and regulations and are reported in accordance with ARRIVE guidelines (https://arriveguidelines.org) for the reporting of animal experiments.

### 2.2 Induction of visual fatigue in rats and grouping

The rats were randomly assigned to four groups, namely normal control (K1) and treatment for 3 days (M1), 7 days (M2), and 14 days (M3), with 10 rats per group. Visual fatigue was induced using levothyroxine sodium gavage combined with tail-clamping stimulation. Briefly, rats in the model groups (M1, M2, and M3) were administered levothyroxine sodium (90 g/kg) by gavage in the morning at 1 mL/100 g. Afterward, the tails of the animals were clamped with a clip (approximately 3 cm from the tip of the tail) for 5 min once a day for effective stimulation to induce the symptoms of liver and kidney yin deficiency. The cages of the rats in the model group were covered with black cloth to simulate a dark environment, and an LED light was placed on the cages as the only light source. The light source was 10–15 cm away from the rat, with an intensity of 3000–500 lx. During the modeling period, the cage was knocked every 15 min to stimulate the rats to keep their eyes open for 2 h per day, and water and food were forbidden.

### 2.3 Sample collection and preparation

At the end of light exposure on day 15, the rats were anesthetized intraperitoneally with 20% urethane and blood was harvested from the abdominal aorta. After standing for 2–4 min, the blood samples were centrifuged at 3000 r/min for 10 min, and the supernatants were isolated and stored at -80°C. Tissues, including the spleen, liver, lens, ciliary body, and eyelids, were rapidly excised. After washing with normal saline, the tissues were ground into a homogenate with a grinding rod in a cold environment and centrifuged at 4°C and 12,000 rpm for 10 min. Finally, supernatants were harvested and stored at -20°C until assayed.

### 2.4 Detection of biochemical parameters

According to the instructions of each kit, the levels of the following indicators were quantified: cyclic adenosine monophosphate (cAMP), cyclic guanosine monophosphate (cGMP), glutathione peroxidase (GSH-Px), total antioxidant capacity (T-AOC), and Ca(2+)/Mg(2+)-ATPase in the lens and ciliary body; aquaporin 4 (AQP4), aquaporin 5 (AQP5), and lactate dehydrogenase (LDH) in the tarsal gland and palpebral muscle; Na(+)/K(+)-ATPase and Ca(2+)/Mg(2+)-ATPase in the liver and cAMP, cGMP, estradiol (E_2_), testosterone (T), corticosterone (CORT), triiodothyronine (T3), thyroxine (T4), interleukin 2 (IL-2), interleukin 6 (IL-6), superoxide dismutase (SOD), and malondialdehyde (MDA).

### 2.5 Untargeted LC-MS/MS metabolomics analysis

#### 2.5.1 Serum sample preprocessing

Serum samples were thawed at 4°C and cold methanol/acetonitrile/water solution was added in the ratio of 2:2:1 (v/v). The mixed samples were vortexed and sonicated for 30 min, followed by incubation at -20°C for 10 min. After centrifugation at 14,000 × g and 4°C for 20 min, the supernatant was harvested and dried under vacuum. For MS analysis, 100 μL of acetonitrile/water solution (1:1, v/v) was added to the sample for re-dissolution, vortexed for 30 min, and centrifuged at 14,000 × g for 15 min. Finally, the supernatant was collected for further analysis.

#### 2.5.2 UHPLC-Q-Exactive orbitrap MS assay

MS separation of the samples was accomplished using a Vanquish LC UHPLC system coupled with a HILIC column. The column temperature was set at 25 °C. Detection was carried out using a binary solvent system with mobile phases A (water containing 25 mM ammonium acetate and 25 mM ammonia) and B (acetonitrile). The specific gradient elution parameters were as follows: 0 to 1.5 min, 98% B; 1.5 to 12 min, 98% B linearly declined to 2%; 12 to 14 min, 2% B; 14 to 14.1 min, 2% B straightly elevated to 98%; 14.1 to 17 min, 98% B. During the analysis period, the temperature of the autoinjector was maintained at 4°C, and the injection volume was 2 μL with a flow rate of 0.3 mL/min.

The MS1 and MS2 spectra were captured using a Q Exactive mass spectrometer, and the chromatographic data were analyzed and detected in the positive (pos) and negative (neg) ion modes of electrospray ionization (ESI). The specific parameters of the ESI source and mass spectrum were as follows: gas 1 and gas 2 were 60; CUR was 30 psi; ion source temperature was 600 °C; spray voltage of positive or negative was 5,500 V; the range of primary mass-to-charge ratio detection was 80–1200 Da, the resolution/secondary resolution was 60,000/30,000, and the cumulative scanning time was 50 ms.

### 2.6 Data preprocessing and analysis

The collected raw data were converted to.mzXML format using ProteoWizard software (23), and XCMS software (24) was utilized for data processing operations, such as peak alignment, retention time correction, and peak area extraction. After matching the raw peak area information with the total ion flow profile, data analysis was conducted. First, the number and classification of the metabolites were identified using the in**-**house database (Shanghai Applied Protein Technology). Next, multivariate statistical analyses were performed to characterize metabolic perturbations and differences among the different groups, including principal component analysis (PCA) and orthogonal partial least squares-discriminant analysis (OPLS-DA). The variable importance for projection (VIP) was calculated based on the OPLS-DA model, which was applied to mine the differential metabolites with biological significance. The selection criterion was VIP > 1 (*P* < 0.05). Finally, to reveal the biological functions of the metabolites, KEGG pathway enrichment analysis was conducted for differential metabolites. Differences between groups were measured using Fisher’s Exact Test, and entries with *P* < 0.05 were selected.

### 2.7 Statistical analysis

Results are expressed as mean ± SEM and were analyzed as well as plotted by GraphPad Prism (version 7.0). Outcomes between the groups were compared using a one-way analysis of variance (ANOVA) and Dunnett’s test. Differences were considered statistically significant at *P* < 0.05.

## 3 Results

### 3.1 Rats with visual fatigue show symptoms of liver and kidney yin deficiency

**Figure 1:**
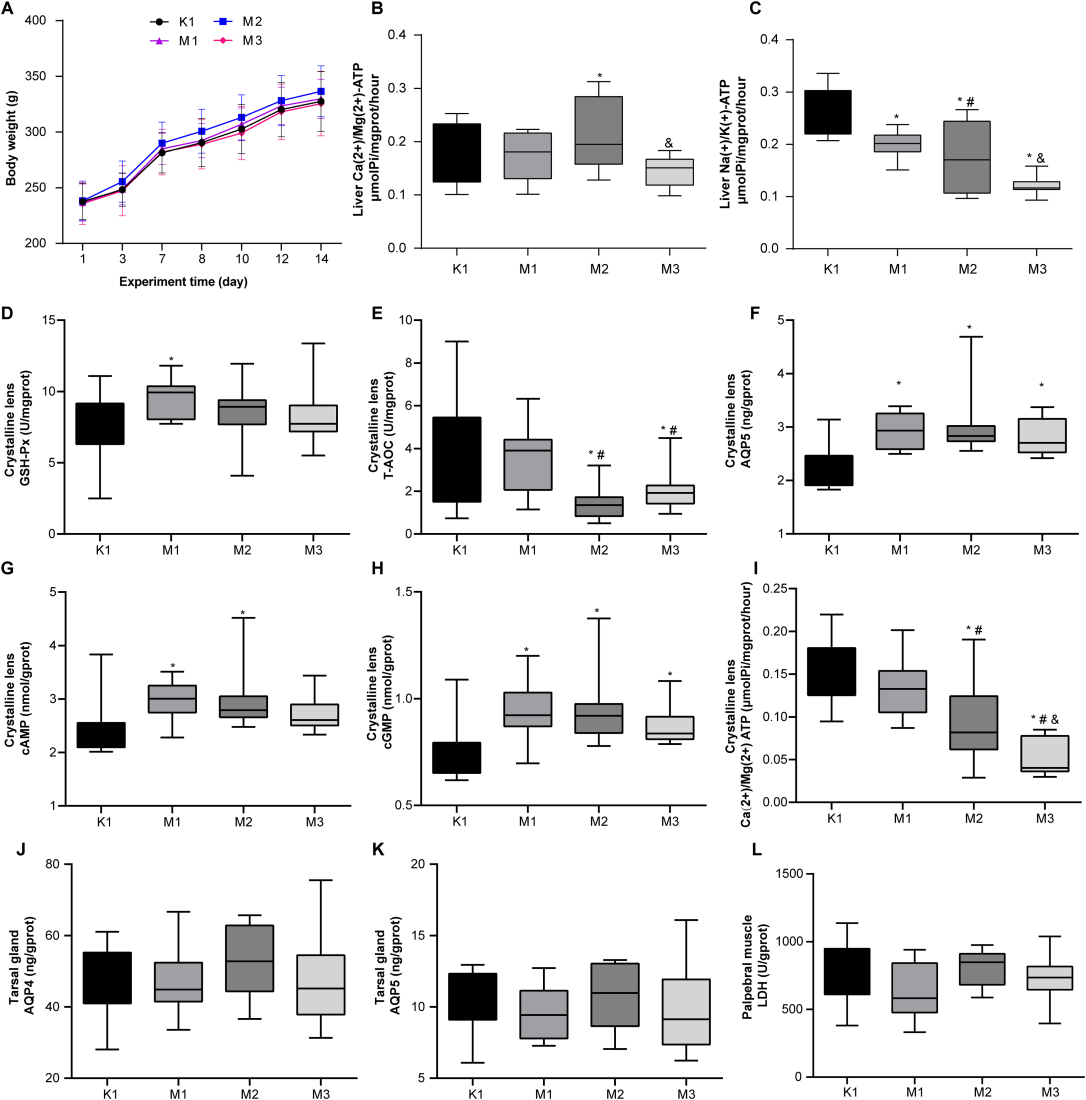
Biochemical and physiological alterations in rats with visual fatigue (n = 10). **(A)** Changes in body weight from day 1 to 14 after visual fatigue induction. **(B)** Liver Ca(2+)/Mg(2+) ATP levels in rats of each group. **(C)** Level of liver Na(+)/K(+) ATP of each group. **(D-I)** Disordered lens-related indexes of GSH - Px **(D)**, T-AOC **(E)**, AQP5 **(F)**, cAMP **(G)**, cGMP **(H)**, and Ca(2+)/Mg(2+) ATP **(I)** in rats with visual fatigue. **(J–L)** Tarsal gland AQP4 **(J)** and AQP5 **(K)** as well as LDH **(L)** levels in palpebral muscles in rats of each group. Data are shown as the mean ± SEM. K1, normal control group; M1, 3-day model group; M2, 7-day model group; M3, 14-day model group. *P < 0.05 vs. K1; ^#^ *P* < 0.05 vs. M1; ^&^ *P* < 0.05 vs. M2.

Throughout the animal experiments (0–14 d), we monitored the body weight changes in all rats and found that the weight of the rats in each group increased slowly, with no significant differences among the four groups (**Figure 1A**). There were no significant differences in the thymus and spleen indices between the K1 and the three model groups (**Supplementary Figures S1A, S1B**). Regarding the liver energy metabolism enzyme activities of rats, compared to the K1 group, the model groups displayed increased Ca(2+)/Mg(2+) ATPase and a decline in Na(+)/K(+) ATPase levels, especially in the M2 group (**Figures 1B, C**). We measured key parameters related to the crystalline lenses and tarsal glands of the rats. The results showed that in the model groups, the levels of GSH-Px, AQP5, cAMP, and cGMP in the lens were elevated, whereas those of T-AOC and Ca(2+)/Mg(2+) ATP were decreased; significant changes in most indicators were observed in the M2 and M3 groups (**Figures 1D–I**). However, the levels of AQP4 and AQP5 in the tarsal gland and LDH in the palpebral muscle did not significantly differ between the normal and model groups (**Figures 1J–L**).

**Figure 2:**
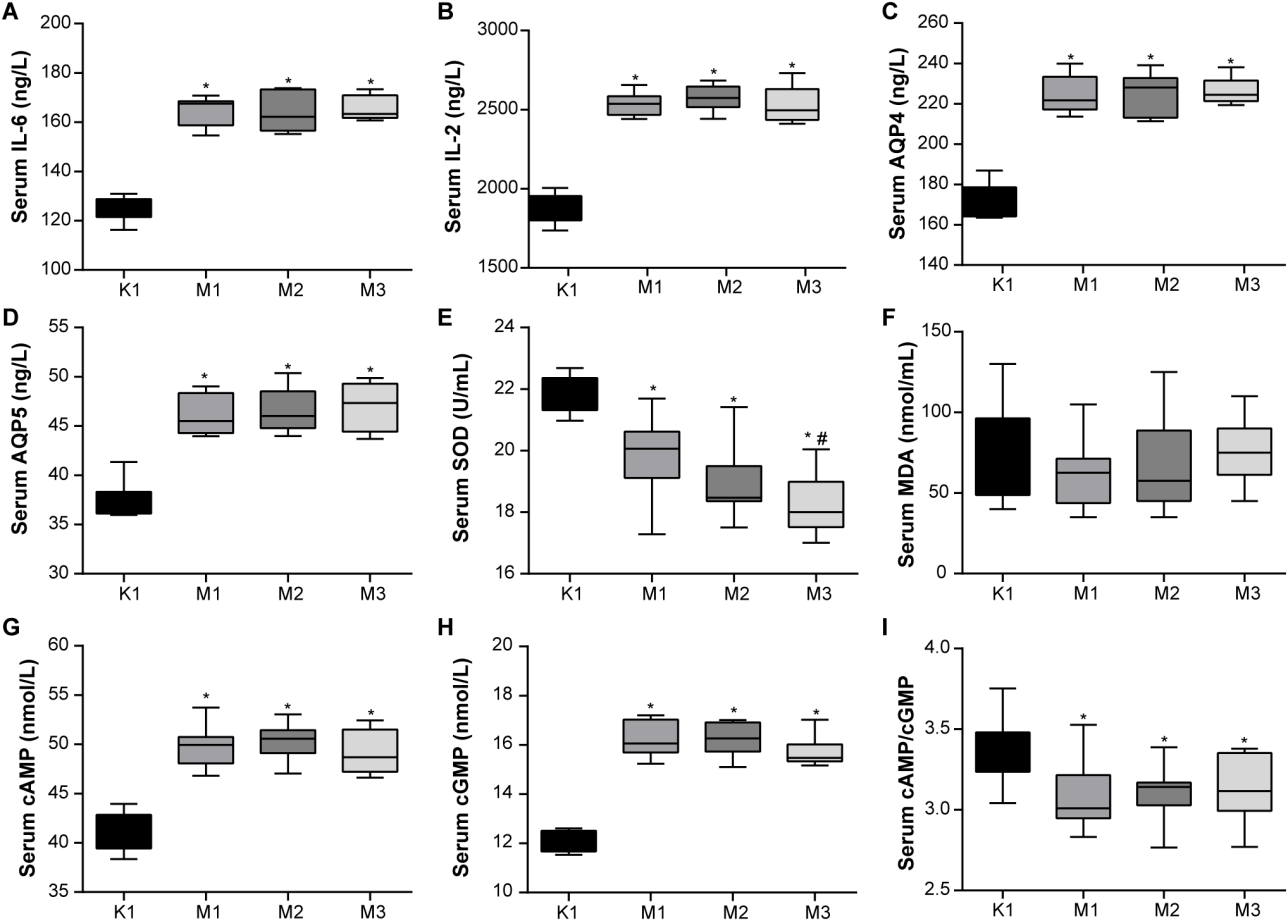
Abnormal blood inflammatory factors and antioxidant activity are observed in rats with visual fatigue (n = 10). **(A)** IL-6. **(B)** IL-2. **(C)** AQP4. **(D)** AQP5. **(E)** SOD. **(F)** MDA. **(G)** cAMP. **(H)** cGMP. **(I)** cAMP/cGMP. Data are shown as the mean ± SEM. K1, normal control group; M1, 3-day model group; M2, 7-day model group; M3, 14-day model group. *P < 0.05 vs. K1; ^#^ *P* < 0.05 vs. M1.

**Figure 3:**
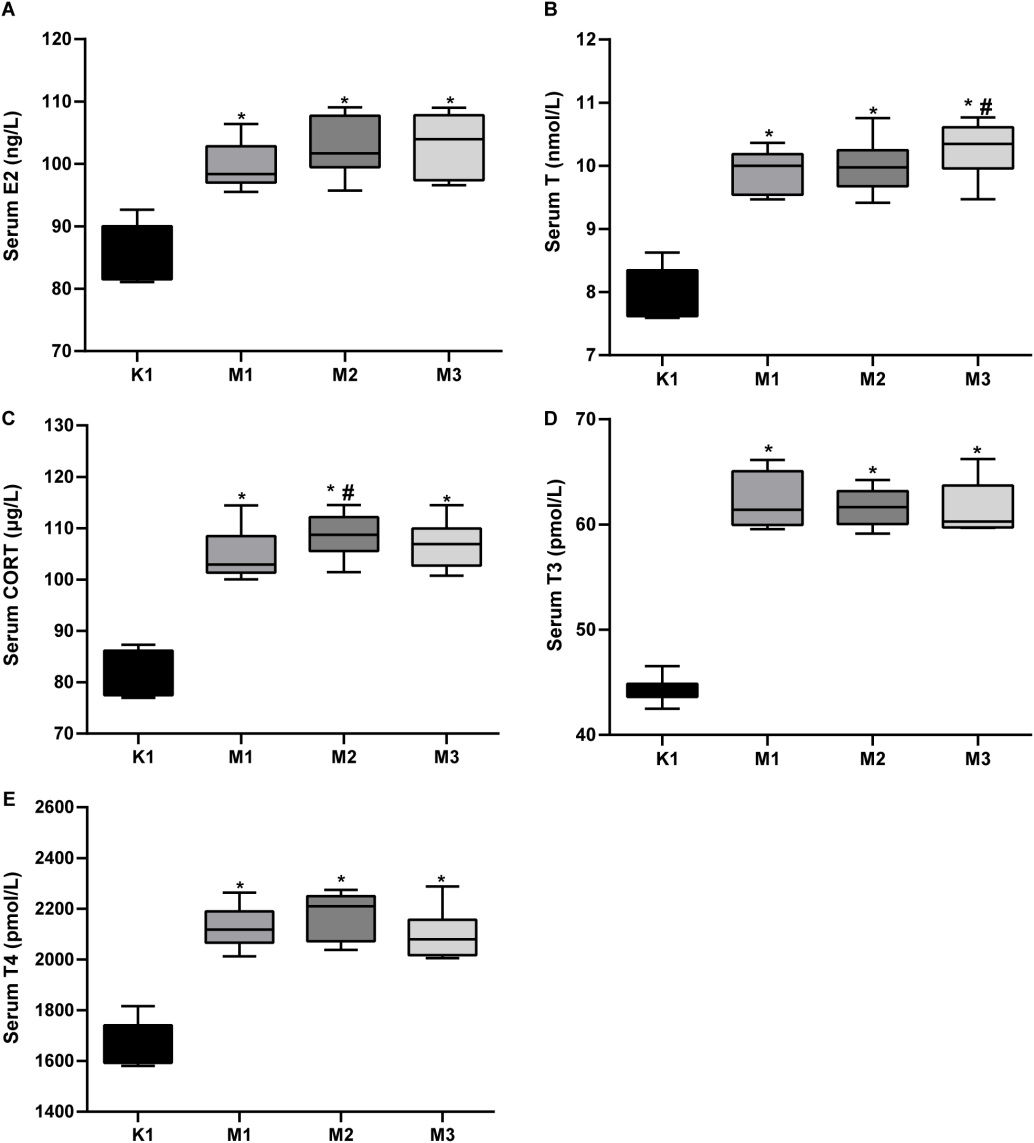
Elevated serum hormones in rats with visual fatigue (n = 10). **(A)** E_2_. **(B)** T. **(C)** CORT. **(D)** T3. **(E)** T4. Data are shown as the mean ± SEM. K1, normal control group; M1, 3-day model group; M2, 7-day model group; M3, 14-day model group. *P < 0.05 vs. K1; ^#^ *P* < 0.05 vs. M1.

Moreover, the levels of inflammatory factors, AQP, antioxidants, and hormones in the serum of rats are important indicators of visual fatigue. As shown in **Figures 2A–I**, the serum of the model groups presented high levels of IL-6, IL-2, AQP4, AQ5, cAMP, and cGMP, while exhibiting low levels of SOD and cAMP/cGMP. However, no significant differences in MDA levels were observed among the four groups. Compared to the normal (K1) group, the levels of serum hormones (E_2_, T, CORT, T3, and T4) increased significantly in rats from the three model groups (**Figures 3A–E**). Overall, these findings indicate that rats in the M2 and M3 groups exhibited both liver and kidney yin deficiency and visual fatigue symptoms, confirming the success of our model construction. The rats in these two groups were selected for further analysis.

### 3.2 Serum metabolic profiles

In the positive and negative ion modes, the spectra of the total ion chromatogram showed that the intensities and retention times of the chromatographic peaks overlapped, indicating that instrument error was negligible (**Supplementary Figure S2A**). PCA revealed that the QC samples in the two modes were closely clustered together, indicating good replicability (**Supplementary Figure S2B**). Overall, the stability, precision, and reproducibility of the UHPLC-Q Exactive Orbitrap MS method were excellent.

### 3.3 Metabolite determination and multivariate statistical analysis

**Figure 4:**
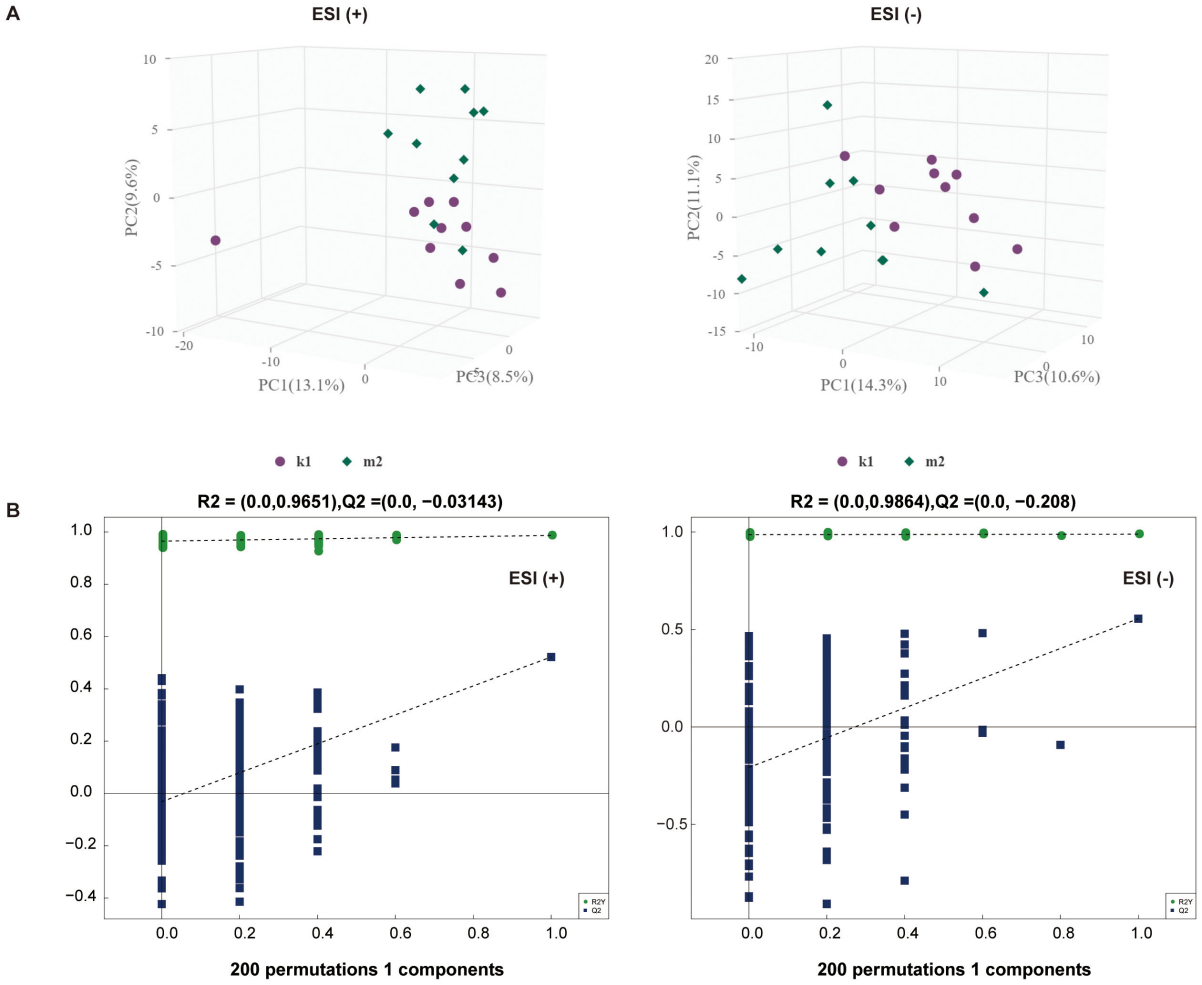
Multivariate statistical analysis for K1 and M2 groups. **(A)** PCA score plot in the positive (left panel) and negative (right panel) modes. **(B)** Permutation test plot of the OPLS-DA model in positive (left panel) and negative (right panel) modes, R^2^ is the explained variance, and Q^2^ is the predictive ability of the model. PC, principal component; ESI, electrospray ionization.

**Figure 5:**
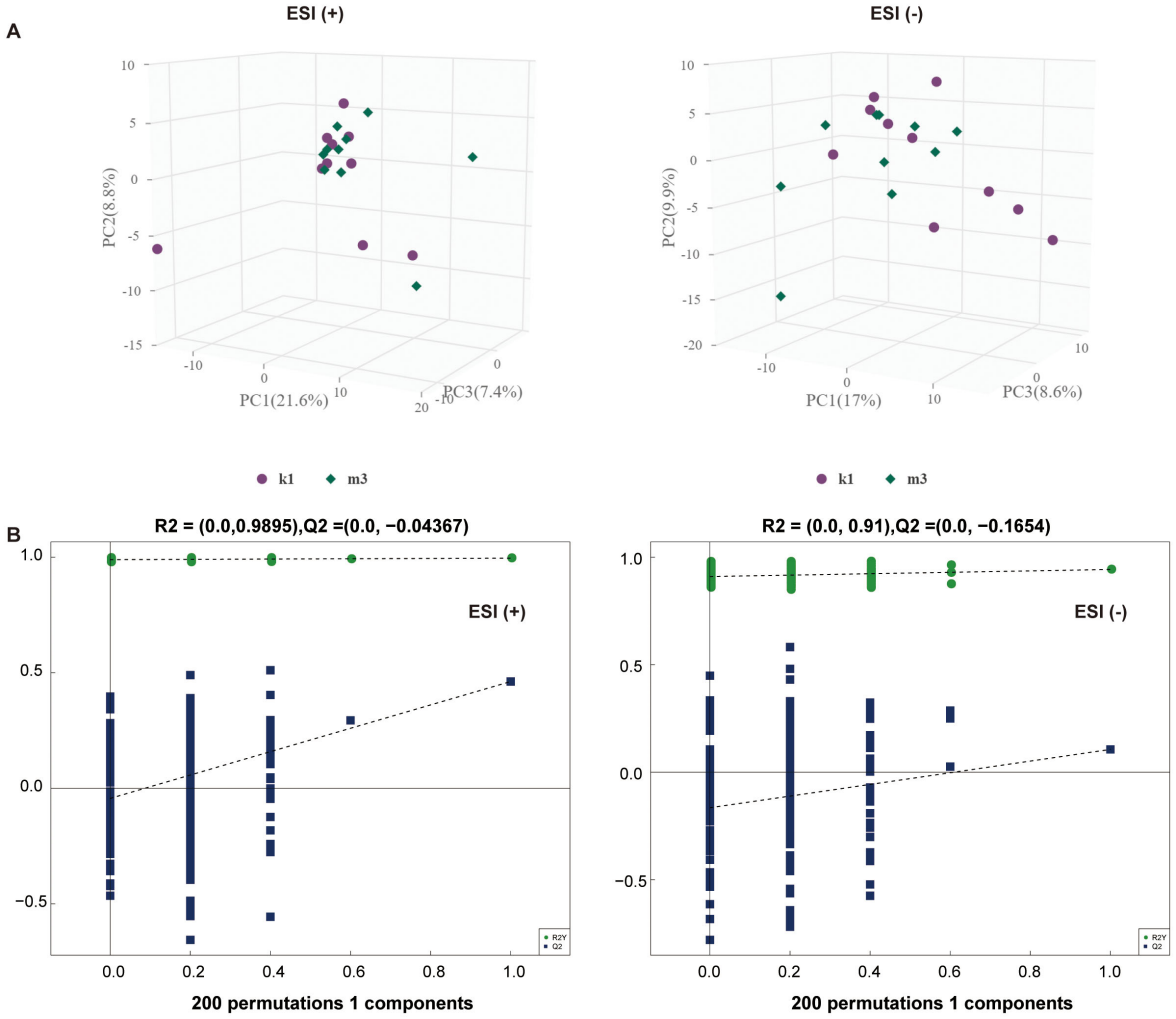
Multivariate statistical analysis for K1 and M3 groups. **(A)** PCA score plot in the positive (left panel) and negative (right panel) modes. **(B)** Permutation test plot of the OPLS-DA model in positive (left panel) and negative (right panel) modes, R^2^ is the explained variance, and Q^2^ is the predictive ability of the model. PC, principal component; ESI, electrospray ionization.

Statistically, 551 (neg) and 695 (pos) metabolites were detected. For M2 *vs*. K1, an examination of the PCA data revealed that the K1 and M2 samples could be clearly separated in both the pos and neg ion modes (**Figure 4A**). An OPLS-DA model was established to further verify the differences between the two groups. The validity of the model was examined using a permutation test. The R2Y/Q2 values for the Pos ions were 0.978/0.522 and 0.989/0.555 for the Neg ion, suggesting that the model was stable and reliable (**Figure 4B**). Similarly, in the comparison between the M3 and K1 groups, we observed a significant separation between the two groups (**Figure 5A**). The R2Y and Q2 parameters of the OPLS-DA model under the pos and neg ion modes were satisfactory (pos: R2Y, 0.996; Q2, 0.462; neg: R2Y, 0.943; Q2, 0.106), implying that the model was not overfitted and that the results were reliable (**Figure 5B**).

### 3.4 Differential metabolite identification

**Figure 6:**
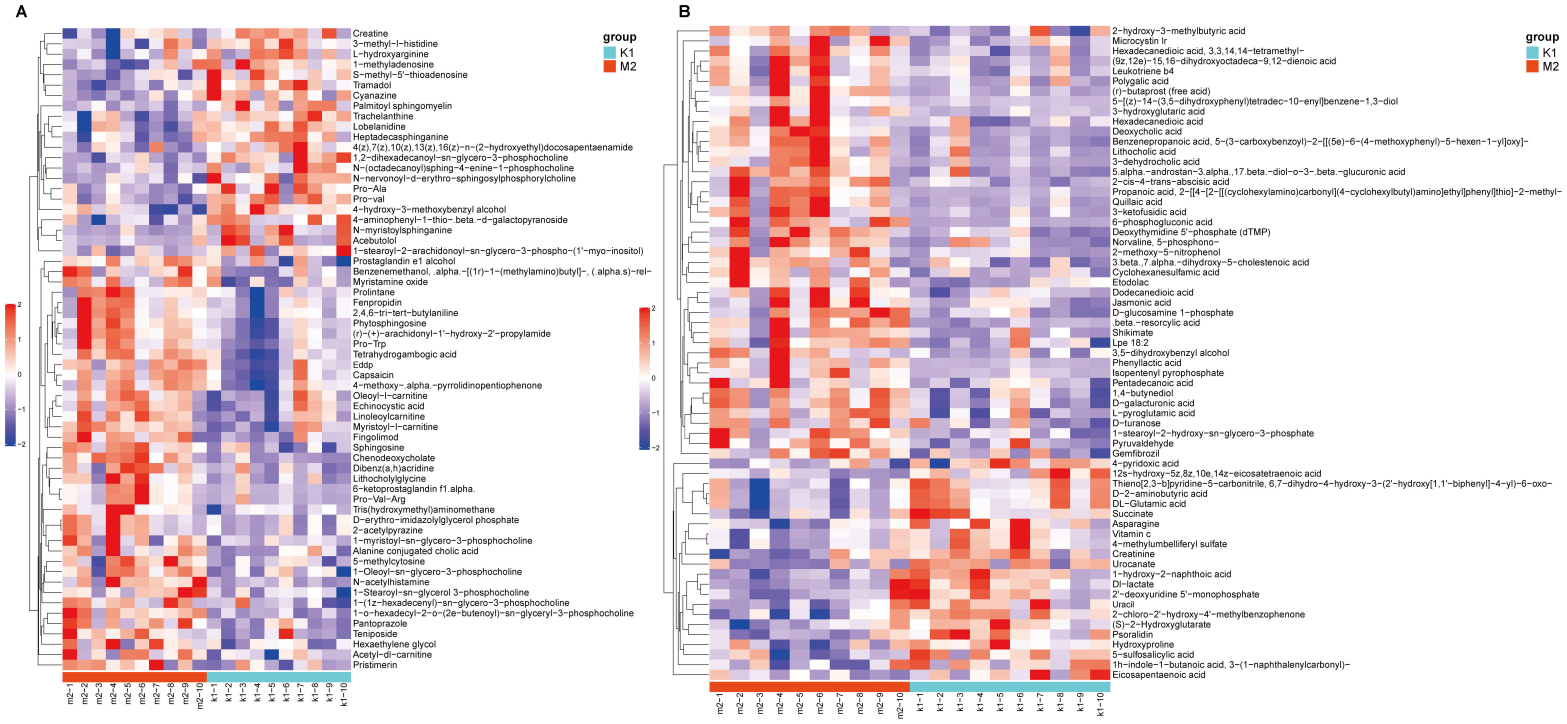
Heatmap of differential metabolites between K1 and M2 groups under the positive **(A)** and negative modes **(B)**. The color red indicates higher relative expression, while purple indicates lower relative expression. K1, normal control group; M2, 7-day model group.

At VIP > 1 and *P* < 0.05, 127 differential metabolites were identified between the K1 and M2 groups, including 62 in Pos and 65 in Neg (**Supplementary Tables S1**, **S2**). In the pos mode, these metabolites were clearly assigned to eight categories, most of which belonged to lipids and lipid-like molecules (21/62, 33.8%), followed by organic nitrogen compounds (9/62, 14.5%) and organic acids and derivatives (9/62, 14.5%). The relative abundances of fingolimod and phytosphingosine were significantly upregulated, whereas that of acebutolol, creatine, and 4-hydroxy-3-methoxybenzyl alcohol were markedly downregulated in the M2 group (**Figure 6A**). In the neg mode, these metabolites belonged to eight classes, primarily lipid-like molecules (23/65, 35.4%) and organic acids and derivatives (13/65, 20%). Notably, in the M2 group, the abundances of 6-phosphogluconic acid and isopentenyl pyrophosphate increased, whereas those of Psoralidin and Urocanate declined (**Figure 6B**).

**Figure 7:**
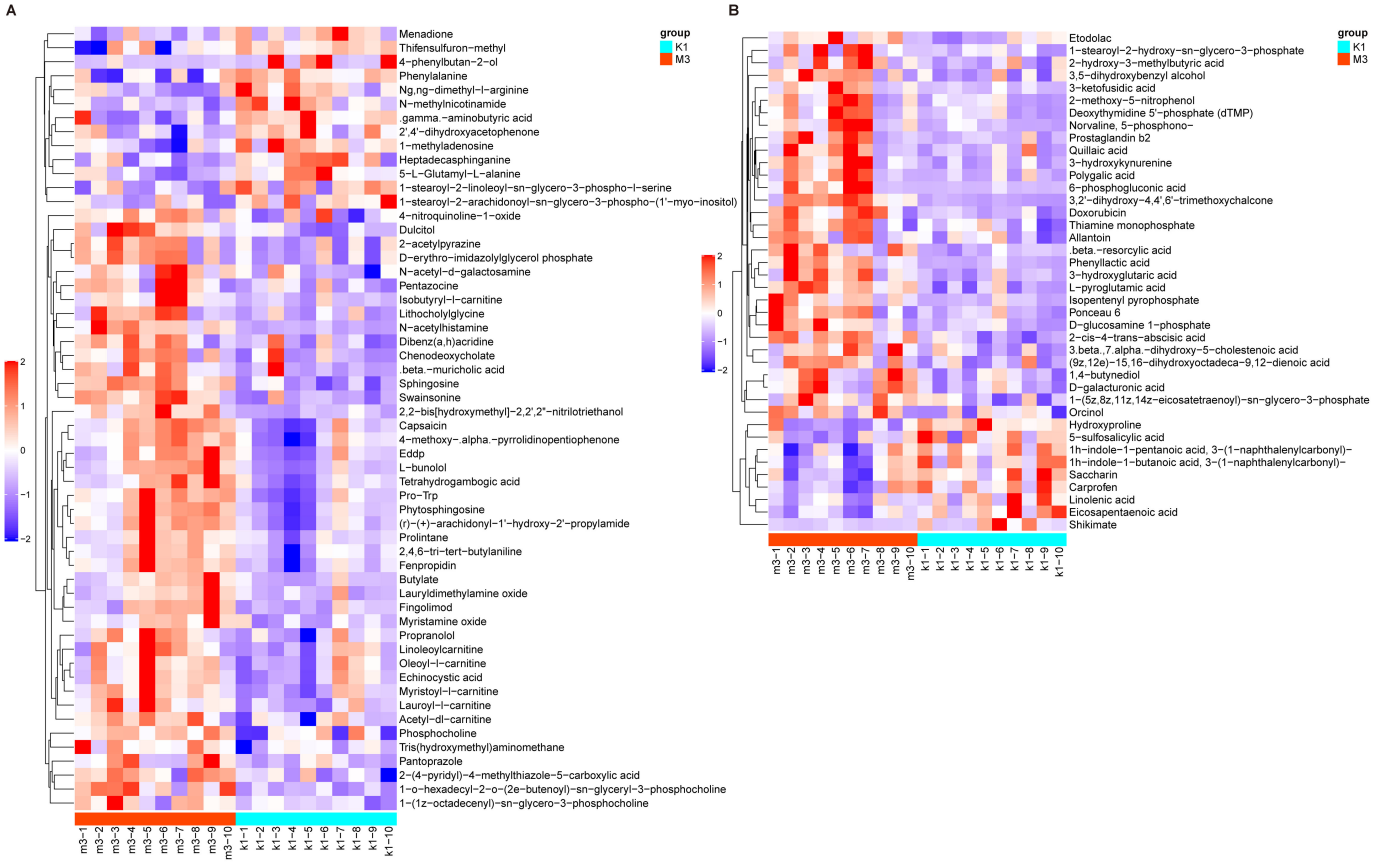
Heatmap of differential metabolites between K1 and M3 groups under the positive **(A)** and negative modes **(B)**. The color red indicates higher relative expression, while purple indicates lower relative expression. K1, normal control group; M3, 14-day model group.

Using the same threshold, 96 differential metabolites were identified between the K1 and M3 groups, with 56 in the Pos and 40 in the Neg modes (**Supplementary Tables S3**, **S4**). In Pos mode, these metabolites were explicitly divided into nine categories– lipids and lipid-like molecules (15/56, 26.8%), organic nitrogen compounds (10/56, 17.9%), and organoheterocyclic compounds (7/56, 12.5%). Compared to the K1 group, 43 metabolites (N-acetylhistamine and fingolimod) were elevated, and 13 metabolites (4−phenylbutan−2−ol and phenylalanine) were reduced in the M3 group (**Figure 7A**). Moreover, in the Neg mode, the 40 metabolites were classified into seven classes– lipids and lipid-like molecules (13/40, 32.5%), organoheterocyclic compounds (6/40, 15%), and organic oxygen compounds (6/40, 15%). We observed that the abundances of 31 metabolites including 6-phosphogluconic acid and 3-ketofusidic acid, increased, whereas those of 9 metabolites, such as shikimate and eicosapentaenoic acid, decreased in the M3 group compared to the K1 group (**Figure 7B**).

### 3.5 Functional pathway enrichment analyses

**Figure 8:**
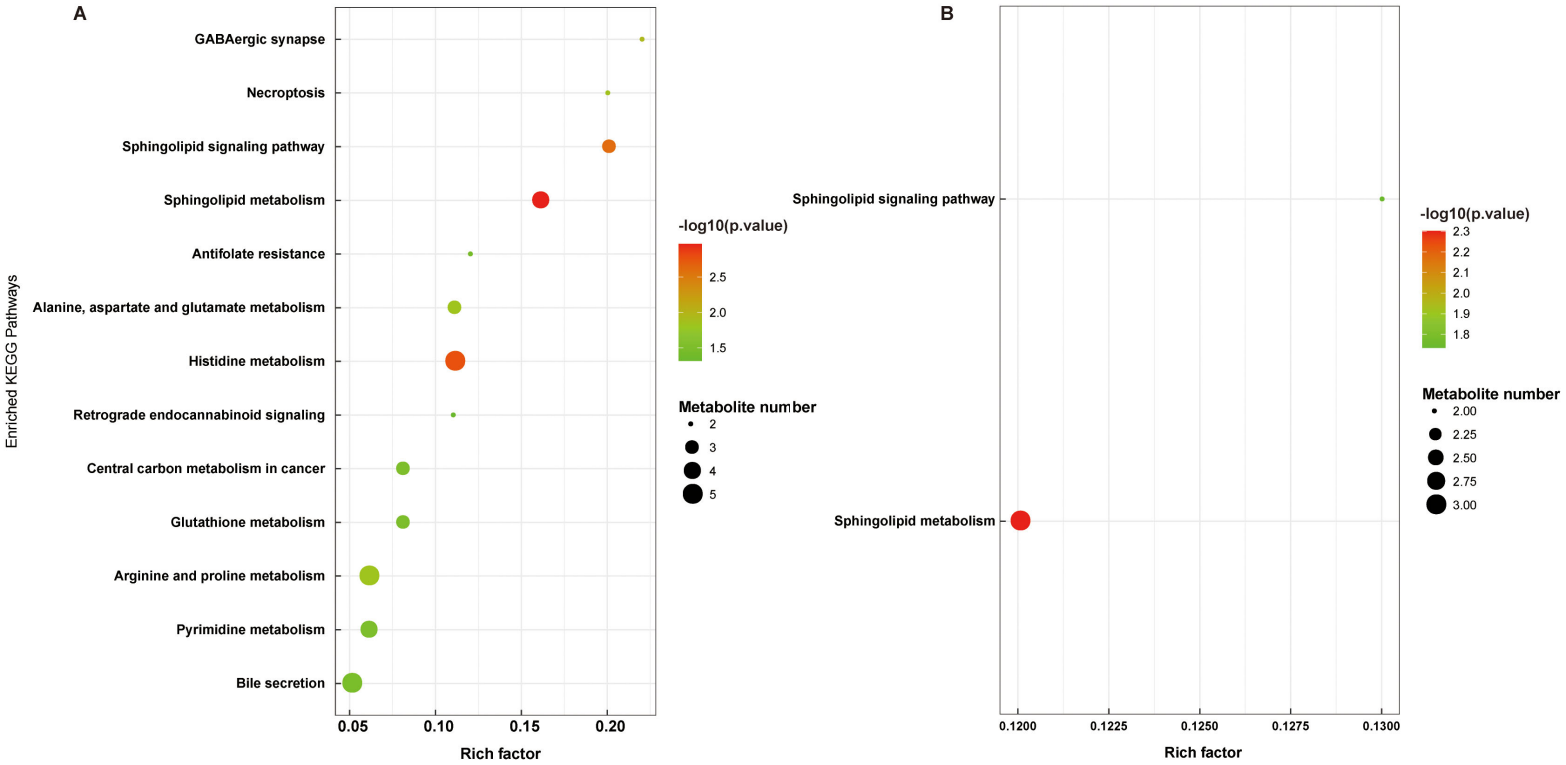
Functional pathway enrichment analyses of differential metabolites between K1 vs. M2 **(A)** and K1 vs. M3 **(B)**. K1, normal control group; M2, 7-day model group; M3, 14-day model group.

To explore the potential metabolic pathways involved related to the differential metabolites, pathway enrichment analysis was conducted. As shown in **Figure 8A**, **B**, the differential metabolites in the K1/M2 and K1/M3 compartments mainly influenced sphingolipid metabolism and the sphingolipid signaling pathway.

## 4 Discussion

Unlike books and printed materials, great information is transmitted through video display terminals, causing increased complains of eye discomfort and fatigue (25). Common symptoms of visual fatigue include visual disturbances, ocular discomfort, and flickering of words within close range. TCM suggests that liver blood depletion and excessive use of the brain can lead to a lack of nourishment for the eyes, thus causing visual fatigue, making liver-kidney yin deficiency evidence of a common visual fatigue pattern. However, little information is available on the development of visual fatigue in the liver-kidney yin deficiency syndrome. The identification of metabolic markers of visual fatigue provides a reference for elucidating the pathological processes and contributes to the development of precise interventions.

In this study, we established animal models of visual fatigue at different time points (3, 7, and 14 d). The results of biochemical indices showed that rats had disorders in the levels of sex hormones and thyroid hormones 3 days after modeling, which confirmed the appearance of liver-kidney yin deficiency. Seven days after modeling, the rats displayed an abnormal metabolism of free radicals, indicating that the visual fatigue model was successfully constructed. These results were confirmed in previous research (22). During the progression of visual fatigue, 127 and 96 significantly abnormal metabolites were identified at 7 and 14 days (compared to controls) after model induction, respectively. Pathway enrichment analysis revealed that these metabolites were involved in sphingolipid metabolism and sphingolipid signaling pathways.

Important causes of visual fatigue include damage to the structure or function of the ocular surface and fundus retina (26). Vision originates from the retina. Damage to the structure of the cells distributed in the retina, especially the retinal pigment epithelium (RPE), is affected (27). RPE cells are extremely active and require large amounts of oxygen to consume energy and nutrients; therefore, they are prone to produce reactive oxygen species (ROS) (28). This process can lead to oxidative stress if ROS are not released in a timely manner (29). On the other hand, continued overuse of the eyes or exposure to abnormal environments (such as bright light) increases the load on the retina and aggravates ocular oxidative stress, leading to dry eyes and eye fatigue (30). Decreased SOD activity has been detected in multiple eye-related diseases, such as cataracts and glaucoma (31, 32), and is regarded as a potential diagnostic marker for oxidative stress-related diseases (33). Reduced SOD activity leads to intense lipid peroxidation (34). Abnormalities in this pathway have been implicated in the pathogenesis of cataracts. Specifically, lipid peroxidation alters the internal composition and conformation of cells by affecting the permeability of the cell membrane, causing loss of protein function, and ultimately leading to cataract formation (35). Moreover, Mg is a cofactor for several enzymes that maintain ionic homeostasis in the lens, such as Ca(2+)-ATPase and Na(+)/K(+) ATPase (36); Mg deficiency impairs ATPase function by enhancing oxidative stress (37). Thus, inhibition of Ca(2+)-ATPase function leads to the loss of calcium homeostasis and calcium accumulation within the lens, which may eventually contribute to lens opacity (38). Consistent with the findings of the above studies, we observed decreased concentrations of serum SOD and lens Ca(2+)-ATPase in the model rats. Accordingly, we speculated that visual fatigue can be alleviated by suppressing oxidative stress and restoring the Ca/Mg balance.

Furthermore, metabolite annotation results showed that the majority of the identified differential metabolites belonged to lipids and lipid-like molecules, organic nitrogen compounds, as well as organic acids and derivatives, such as fingolimod, phytosphingosine, sphingosine, quillaic acid, phenylalanine and creatine. These metabolites also affect sphingolipid metabolism and the sphingolipid signaling pathway during liver and kidney yin deficiency - type visual fatigue progression. With the deepening of our understanding of ophthalmic diseases, numerous studies have revealed that lipid and sphingolipid signaling pathways, as well as organic nitrogen compounds and organic acids, are involved in the pathological mechanisms of ocular inflammatory diseases (39–41). Among the identified differential metabolites, organic acids are a significant proportion. For example, phenylalanine is an essential amino acid and neurotransmitter precursor, and creatine plays a key role in cellular energy metabolism and antioxidant defense. This study observed reduced levels of phenylalanine and creatine in the model group. Combined with previous research reports, phenylalanine modification may reduce empty capsids to lower virus - vector - induced intraocular inflammatory responses (42). Creatine supplementation may delay cone secondary degeneration in retinitis pigmentosa and protect the retina by reducing oxidative stress and inflammation (43). Thus, maintaining or restoring the homeostasis of these organic acids may help delay visual fatigue progression.

Lipids, especially sphingolipids, are enriched in the nervous system and retina (44). Sphingolipid metabolism disorders are associated with visual dysfunction such as visual fatigue. For example, Green et al. found that patients with hereditary neuroretinas exhibit altered levels of complex sphingolipids (45). This study detected abnormal expression of the organic nitrogen compound metabolite fingolimod. Fingolimod (also known as FTY720, a sphingosine analog) is used clinically as an S1PR agonist and immunomodulatory drug for the treatment of recurrent multiple sclerosis (46, 47). Interestingly, multiple sclerosis presents symptoms such as visual impairment and fatigue (48). Fingolimod/FTY720 is found to inhibit NFκB and pro-inflammatory cytokines in the retina of diabetic rats, preventing disruption of the blood-retinal barrier; in addition, FTY720 reduces neuronal loss in rats with glaucoma (49, 50). In TCM theory, liver and kidney yin deficiency may relate to endocrine and immune disorders (51). As an immunomodulatory agent, we speculate that fingolimod may participate in the pathological process of liver and kidney yin deficiency type visual fatigue by regulating ocular immune response or neuroprotective mechanisms. Despite its promising applications, fingolimod has been documented to cause possible adverse ocular effects (such as macular edema) (52). Therefore, further studies are needed to confirm and evaluate the role of fingolimod in improving visual function disorders like visual fatigue. Porter et al. (53) showed that the sphingosine 1-phosphate signaling pathway is implicated in many key processes in the eye, ranging from light stress/apoptotic responses to retinal/vascular development, suggesting that the sphingolipid signaling pathway serves a role in both vision and apoptosis. Another differential metabolite, quillaic acid, exhibits various biological activities, including anti-inflammatory, antiviral, and antineoplastic effects (54, 55). It also exhibits the ability to induce apoptosis in various cell types. Although there is currently no direct evidence of quillaic acid’s role in visual fatigue, based on its known anti-inflammatory properties, we speculate that it may be beneficial in alleviating liver and kidney yin deficiency - induced visual fatigue symptoms. Taken together, the key differential metabolites and disrupted sphingolipid metabolic pathways found in this study’s liver and kidney yin deficiency visual fatigue model suggest that they may contribute to visual fatigue development by affecting biological processes such as retinal inflammation and apoptosis. This provides a new perspective on understanding metabolic characteristics of visual fatigue, innovatively establishing a link between TCM syndromes and sphingolipid metabolic disorders, revealing the complementarity between the traditional TCM syndrome classification system and modern neuro - ophthalmic cognition. However, further research is needed to explore the specific molecular mechanisms.

Information on the signature metabolites associated with liver and kidney yin deficiency patterns in visual fatigue is limited, and our work fills this gap. The screened metabolites contribute to understanding the underlying causes of visual fatigue. However, this study has several limitations. First, the inherent physiological differences between rats and humans restrict the direct extrapolation of results. Second, the assessment of visual fatigue and liver-kidney yin deficiency in the animal model mainly depends on biochemical indicators. Future research should integrate more objective behavioral evaluations, such as optokinetic responses and pupil reaction, for a comprehensive visual function assessment. Moreover, the untargeted metabolomics analysis used here is exploratory. The identified differential metabolites future need quantitative validation and mechanistic exploration through targeted metabolomics (e.g., LC-MS/MS) or functional experiments. Therefore, the results presented here should be interpreted with caution. Finally, the mechanisms of sphingolipid metabolism and signaling pathways implicated in visual fatigue need to be further explored in large populations.

## 5 Conclusions

Overall, we revealed the metabolic profiles of an animal model of visual fatigue with liver and kidney yin deficiency syndrome and identified metabolites and signaling pathways prominently associated with this disease. Sphingolipid metabolites are expected to be potential targets for alleviating visual fatigue. Further targeted metabolomics or functional studies are needed to quantitative validation our findings, confirm metabolite targets, and address animal model limitations. This study offers fresh insights into visual fatigue-related metabolic signatures, bridges TCM theory with modern neuro-ophthalmological metabolic mechanisms, and lays an initial scientific foundation for precise disease intervention.

## Data Availability

The original contributions presented in the study are included in the article/**Supplementary Material**. Further inquiries can be directed to the corresponding author/s.

## Supplementary material

Supplementary Figure 1 Changes in thymus index **(A)** and spleen index **(B)** in rats in each group (n = 10). K1, normal control group; M1, 3-day model group; M2, 7-day model group; M3, 14-day model group.

Supplementary Figure 2 Peak intensity chromatograms of serum samples in positive and negative modes. **(A)**: Total ion chromatogram. **(B)**: Metabolic profiles of the serum samples in the PCA plot. PCA, principal component analysis; ESI, electrospray ionization.

Supplementary Table 1 List of differential metabolites between K1 and M2 groups under the positive mode.

Supplementary Table 2 List of differential metabolites between K1 and M2 groups under the negative mode.

Supplementary Table 3 List of differential metabolites between K1 and M3 groups under the positive mode.

Supplementary Table 4 List of differential metabolites between K1 and M3 groups under the negative mode.

## Glossary

MS: mass spectrometry
TCM: traditional Chinese medicine
SD: Sprague-Dawley
SPF: specific pathogen-free
cAMP: cyclic adenosine monophosphate
cGMP: cyclic guanosine monophosphate
GSH-Px: glutathione peroxidase
T-AOC: total antioxidant capacity
AQP4: aquaporin 4
AQP5: aquaporin 5
LDH: lactate dehydrogenase
E2: estradiol
T: testosterone
CORT: corticosterone
T3: triiodothyronine
T4: thyroxine
IL-2: interleukin 2
IL-6: interleukin 6
SOD: superoxide dismutase
MDA: malondialdehyde
pos: positive
neg: negative
ESI: electrospray ionization
PCA: principal component analysis
OPLS-DA: orthogonal partial least squares-discriminant analysis
VIP: variable importance for projection
ANOVA: analysis of variance
RPE: retinal pigment epithelium
ROS: reactive oxygen species.

## References

[1] Le Z Antonov E Mao Q Petrov V Wang Y Wang W. Anti-fatigue glasses based on microprisms for preventing eyestrain. Sensors (Basel). (2022) 22:1933. doi: 10.3390/s22051933, PMID: 35271080 PMC8914742

[2] Simonson E Brozek J Keys A. Visual fatigue. Fed Proc. (1947) 6:202. 20244254

[3] Ip JM Robaei D RochtChina E Mitchell P. Prevalence of eye disorders in young children with eyestrain complaints. Am J Ophthalmol. (2006) 142:495–7. doi: 10.1016/j.ajo.2006.03.047, PMID: 16935600

[4] Vilela MAP Pellanda LC Fassa AG Castagno VD. Prevalence of asthenopia in children: a systematic review with meta-analysis. J Pediatr (Rio J). (2015) 91:320–5. doi: 10.1016/j.jped.2014.10.008, PMID: 25986614

[5] Zheng F Hou F Chen R Mei J Huang P Chen B. Investigation of the relationship between subjective symptoms of visual fatigue and visual functions. Front Neurosci. (2021) 15:686740. doi: 10.3389/fnins.2021.686740, PMID: 34335163 PMC8319646

[6] Jaiswal S Asper L Long J Lee A Harrison K Golebiowski B. Ocular and visual discomfort associated with smartphones, tablets and computers: what we do and do not know. Clin Exp Optom. (2019) 102:463–77. doi: 10.1111/cxo.12851, PMID: 30663136

[7] Aakre BM Doughty MJ. Are there differences between ‘visual symptoms’ and specific ocular symptoms associated with video display terminal (VDT) use? Cont Lens Anterior Eye. (2007) 30:174–82. doi: 10.1016/j.clae.2007.01.001, PMID: 17293157

[8] Pang L. Ancient Chinese medical books on visual fatigue and its syndrome and treatment. J Basic Chin Med. (2007) 133:669. doi: 10.3969/j.issn.1006-3250.2007.09.015

[9] Shi G Chen S. Progress in traditional Chinese medicine treatment of video terminal visual fatigue. Asia-Pacific Traditional Med. (2019) 15:190–2. doi: 10.11954/ytctyy.201909059

[10] Zeng J Wang M Huang Y Yan J. Analysis of visual fatigue in patients with symptoms and syndrome type. Med Inf. (2015) 28:42–3. doi: 10.3969/j.issn.1006-1959.2015.01.056

[11] Huang Z Zhang J Zhao D Zhou X Wang C Chen Y. Establishment of a mice model of the yin deficiency pattern of the liver and kidney and the effect of Gouqi Juhua Fang on asthenopia. J Beijing Univ Traditional Chin Med. (2022) 45:578–86. doi: 10.3969/j.issn.1006-2157.2022.06.022

[12] Muthubharathi BC Gowripriya T Balamurugan K. Metabolomics: small molecules that matter more. Mol Omics. (2021) 17:210–29. doi: 10.1039/D0MO00176G, PMID: 33598670

[13] Flam E Haas JT Staels B. Liver metabolism in human MASLD: A review of recent advancements using human tissue metabolomics. Atherosclerosis. (2025) 400:119054. doi: 10.1016/j.atherosclerosis.2024.119054, PMID: 39586140

[14] Singh P Singh R Pasricha C Kumari P. Navigating liver health with metabolomics: A comprehensive review. Clin Chim Acta. (2025) 566:120038. doi: 10.1016/j.cca.2024.120038, PMID: 39536895

[15] Pandey S. Metabolomics for the identification of biomarkers in kidney diseases. Nanotheranostics. (2025) 9:110–20. doi: 10.7150/ntno.108320, PMID: 40212952 PMC11980039

[16] Ragi N Sharma K. Deliverables from metabolomics in kidney disease: adenine, new insights, and implication for clinical decision-making. Am J Nephrol. (2024) 55:421–38. doi: 10.1159/000538051, PMID: 38432206

[17] Wang T Liu J Luo X Hu L Lu H. Functional metabolomics innovates therapeutic discovery of traditional Chinese medicine derived functional compounds. Pharmacol Ther. (2021) 224:107824. doi: 10.1016/j.pharmthera.2021.107824, PMID: 33667524

[18] Zhao M Che Y Gao Y Zhang X. Application of multi-omics in the study of traditional Chinese medicine. Front Pharmacol. (2024) 15:1431862. doi: 10.3389/fphar.2024.1431862, PMID: 39309011 PMC11412821

[19] Ren X Jiang S Lu Y Luo X Guo H Miao M. Study on the relationship between liver-kidney yin deficiency and blood lipid, energy metabolism, as well as free radical reaction. Henan Traditional Chin Med. (1996) 16:20–2.

[20] Giannaccare G Pellegrini M Senni C Bernabei F Scorcia V Cicero AFG. Clinical applications of astaxanthin in the treatment of ocular diseases: emerging insights. Mar Drugs. (2020) 18:239. doi: 10.3390/md18050239, PMID: 32370045 PMC7281326

[21] Li X Wang P Tong Y Liu J Shu G. UHPLC-Q-exactive orbitrap MS/MS-based untargeted metabolomics and molecular networking reveal the differential chemical constituents of the bulbs and flowers of fritillaria thunbergii. Molecules. (2022) 27:6944. doi: 10.3390/molecules27206944, PMID: 36296537 PMC9609367

[22] Zhao D Zhang J Huang Z Zhou X Wang C Wang L. Establishment of visual fatigue model of liver-kidney yin deficiency combined with blue light irradiation and efficacy evaluation of Qiju Mingmu Tablets. China J Traditional Chin Med Pharm. (2021) 36:2044–9.

[23] Kessner D Chambers M Burke R Agus D Mallick P. ProteoWizard: open source software for rapid proteomics tools development. Bioinformatics. (2008) 24:2534–6. doi: 10.1093/bioinformatics/btn323, PMID: 18606607 PMC2732273

[24] Mahieu NG Genenbacher JL Patti GJ. A roadmap for the XCMS family of software solutions in metabolomics. Curr Opin Chem Biol. (2016) 30:87–93. doi: 10.1016/j.cbpa.2015.11.009, PMID: 26673825 PMC4831061

[25] Tang Y Wei X Huang Y Huang J Jin W Lu Y. Intervention effect of traditional chinese medicine hot pressing combined with health education on the adolescent’s visual fatigue. J Healthc Eng. (2022) 2022:2450197. doi: 10.1155/2022/2450197, PMID: 35360485 PMC8964189

[26] Ayaki M Kuze M Kondo M Tsubota K Negishi K. Association between retinal nerve fiber layer thickness and eye fatigue. BioMed Res Int. (2019) 2019:3014567. doi: 10.1155/2019/3014567, PMID: 30809534 PMC6364103

[27] Yang S Zhou J Li D. Functions and diseases of the retinal pigment epithelium. Front Pharmacol. (2021) 12:727870. doi: 10.3389/fphar.2021.727870, PMID: 34393803 PMC8355697

[28] Chan C-M Huang D-Y Sekar P Hsu S-H Lin W-W. Reactive oxygen species-dependent mitochondrial dynamics and autophagy confer protective effects in retinal pigment epithelial cells against sodium iodate-induced cell death. J BioMed Sci. (2019) 26:40. doi: 10.1186/s12929-019-0531-z, PMID: 31118030 PMC6532221

[29] Apel K Hirt H. Reactive oxygen species: metabolism, oxidative stress, and signal transduction. Annu Rev Plant Biol. (2004) 55:373–99. doi: 10.1146/annurev.arplant.55.031903.141701, PMID: 15377225

[30] Portello JK Rosenfield M Chu CA. Blink rate, incomplete blinks and computer vision syndrome. Optom Vis Sci. (2013) 90:482–7. doi: 10.1097/OPX.0b013e31828f09a7, PMID: 23538437

[31] Babizhayev MA. Mitochondria induce oxidative stress, generation of reactive oxygen species and redox state unbalance of the eye lens leading to human cataract formation: disruption of redox lens organization by phospholipid hydroperoxides as a common basis for cataract disease. Cell Biochem Funct. (2011) 29:183–206. doi: 10.1002/cbf.1737, PMID: 21381059

[32] Li S Shao M Li Y Li X Wan Y Sun X. Relationship between oxidative stress biomarkers and visual field progression in patients with primary angle closure glaucoma. Oxid Med Cell Longev. (2020) 2020:2701539. doi: 10.1155/2020/2701539, PMID: 32831992 PMC7428947

[33] Hsueh Y Chen Y Tsao Y Cheng C Wu W Chen H. The pathomechanism, antioxidant biomarkers, and treatment of oxidative stress-related eye diseases. Int J Mol Sci. (2022) 23:1255. doi: 10.3390/ijms23031255, PMID: 35163178 PMC8835903

[34] Balmus I-M Alexa AI Ciuntu R-E Danielescu C Stoica B Cojocaru SI. Oxidative stress markers dynamics in keratoconus patients’ tears before and after corneal collagen crosslinking procedure. Exp Eye Res. (2020) 190:107897. doi: 10.1016/j.exer.2019.107897, PMID: 31836491

[35] Miric DJ Kisic BM Zoric LD Miric BM Mirkovic M Mitic R. Influence of cataract maturity on aqueous humor lipid peroxidation markers and antioxidant enzymes. Eye (Lond). (2014) 28:72–7. doi: 10.1038/eye.2013.207, PMID: 24097121 PMC3890749

[36] Umeda IO Kashiwa Y Nakata H Nishigori H. Predominant phosphatase in the ocular lens regulated by physiological concentrations of magnesium and calcium. Life Sci. (2003) 73:1161–73. doi: 10.1016/S0024-3205(03)00379-5, PMID: 12818724

[37] Nagai N Fukuhata T Ito Y. Effect of magnesium deficiency on intracellular ATP levels in human lens epithelial cells. Biol Pharm Bull. (2007) 30:6–10. doi: 10.1248/bpb.30.6, PMID: 17202650

[38] Iezhitsa I Agarwal R Saad SDB Zakaria FKB Agarwal P Krasilnikova A. Mechanism of the anticataract effect of liposomal MgT in galactose-fed rats. Mol Vis. (2016) 22:734–47., PMID: 27440992 PMC4942261

[39] Grambergs R Mondal K Mandal N. Inflammatory ocular diseases and sphingolipid signaling. Adv Exp Med Biol. (2019) 1159:139–52. doi: 10.1007/978-3-030-21162-2_8, PMID: 31502203 PMC8655850

[40] Nicholas SE Rowsey TG Priyadarsini S Mandal NA Karamichos D. Unravelling the interplay of sphingolipids and TGF-β signaling in the human corneal stroma. PLoS One. (2017) 12:e0182390. doi: 10.1371/journal.pone.0182390, PMID: 28806736 PMC5555661

[41] She X Zhou Y Liang Z Wei J Xie B Zhang Y. Metabolomic study of a rat model of retinal detachment. Metabolites. (2022) 12(11):1077. doi: 10.3390/metabo12111077, PMID: 36355160 PMC9699637

[42] Timmers AM Newmark JA Turunen HT Farivar T Liu J Song C. Ocular inflammatory response to intravitreal injection of adeno-associated virus vector: relative contribution of genome and capsid. Hum Gene Ther. (2020) 31:80–9. doi: 10.1089/hum.2019.144, PMID: 31544533

[43] Narayan DS Chidlow G Wood JPM Casson RJ. Investigations into bioenergetic neuroprotection of cone photoreceptors: relevance to retinitis pigmentosa. Front Neurosci. (2019) 13:1234. doi: 10.3389/fnins.2019.01234, PMID: 31803010 PMC6872495

[44] Shiwani HA Elfaki MY Memon D Ali S Aziz A Egom EE. Updates on sphingolipids: Spotlight on retinopathy. BioMed Pharmacother. (2021) 143:112197. doi: 10.1016/j.biopha.2021.112197, PMID: 34560541

[45] Green CR Bonelli R Ansell BRE Tzaridis S Handzlik MK McGregor GH. Divergent amino acid and sphingolipid metabolism in patients with inherited neuro-retinal disease. Mol Metab. (2023) 72:101716. doi: 10.1016/j.molmet.2023.101716, PMID: 36997154 PMC10114224

[46] Brinkmann V Billich A Baumruker T Heining P Schmouder R Francis G. Fingolimod (FTY720): discovery and development of an oral drug to treat multiple sclerosis. Nat Rev Drug Discov. (2010) 9:883–97. doi: 10.1038/nrd3248, PMID: 21031003

[47] Huwiler A Zangemeister-Wittke U. The sphingosine 1-phosphate receptor modulator fingolimod as a therapeutic agent: Recent findings and new perspectives. Pharmacol Ther. (2018) 185:34–49. doi: 10.1016/j.pharmthera.2017.11.001, PMID: 29127024

[48] Abdel Naseer M Shehata HS Khalil S Fouad AM Abdelghany H. Prevalence of primary headaches in multiple sclerosis patients. Mult Scler Relat Disord. (2024) 86:105602. doi: 10.1016/j.msard.2024.105602, PMID: 38598953

[49] Fan L Yan H. FTY720 attenuates retinal inflammation and protects blood-retinal barrier in diabetic rats. Invest Ophthalmol Vis Sci. (2016) 57:1254–63. doi: 10.1167/iovs.15-18658, PMID: 26986045

[50] You Y Gupta VK Li JC Al-Adawy N Klistorner A Graham SL. FTY720 protects retinal ganglion cells in experimental glaucoma. Invest Ophthalmol Vis Sci. (2014) 55:3060–6. doi: 10.1167/iovs.13-13262, PMID: 24744204

[51] Yu J Yu CQ Cao Q Wang L Wang WJ Zhou LR. Consensus on the integrated traditional Chinese and Western medicine criteria of diagnostic classification in polycystic ovary syndrome (draft). J Integr Med. (2017) 15:102–9. doi: 10.1016/S2095-4964(17)60331-5, PMID: 28285615

[52] Mandal P Gupta A Fusi-Rubiano W Keane PA Yang Y. Fingolimod: therapeutic mechanisms and ocular adverse effects. Eye (Lond). (2017) 31:232–40. doi: 10.1038/eye.2016.258, PMID: 27886183 PMC5306460

[53] Porter H Qi H Prabhu N Grambergs R McRae J Hopiavuori B. Characterizing sphingosine kinases and sphingosine 1-phosphate receptors in the mammalian eye and retina. Int J Mol Sci. (2018) 19:3885. doi: 10.3390/ijms19123885, PMID: 30563056 PMC6321283

[54] Arrau S Delporte C Cartagena C Rodríguez-Díaz M González P Silva X. Antinociceptive activity of Quillaja saponaria Mol. saponin extract, quillaic acid and derivatives in mice. J Ethnopharmacol. (2011) 133:164–7. doi: 10.1016/j.jep.2010.09.016, PMID: 20951193

[55] Arrau S Rodríguez-Díaz M Cassels BK Valenzuela-Barra G Delporte C Barriga A. Antihyperalgesic activity of quillaic acid obtained from quillaja saponaria mol. Curr Top Med Chem. (2019) 19:927–30. doi: 10.2174/1568026619666190509115741, PMID: 31072292

