# Supplementary figures and images for: Metabolomic profiling in a rat model of visual fatigue associated with liver-kidney yin deficiency syndrome
Source: Front Endocrinol (Lausanne). 2025 Jul 28;16:1586581. doi: 10.3389/fendo.2025.1586581 (PMC12336029; doi:10.3389/fendo.2025.1586581)

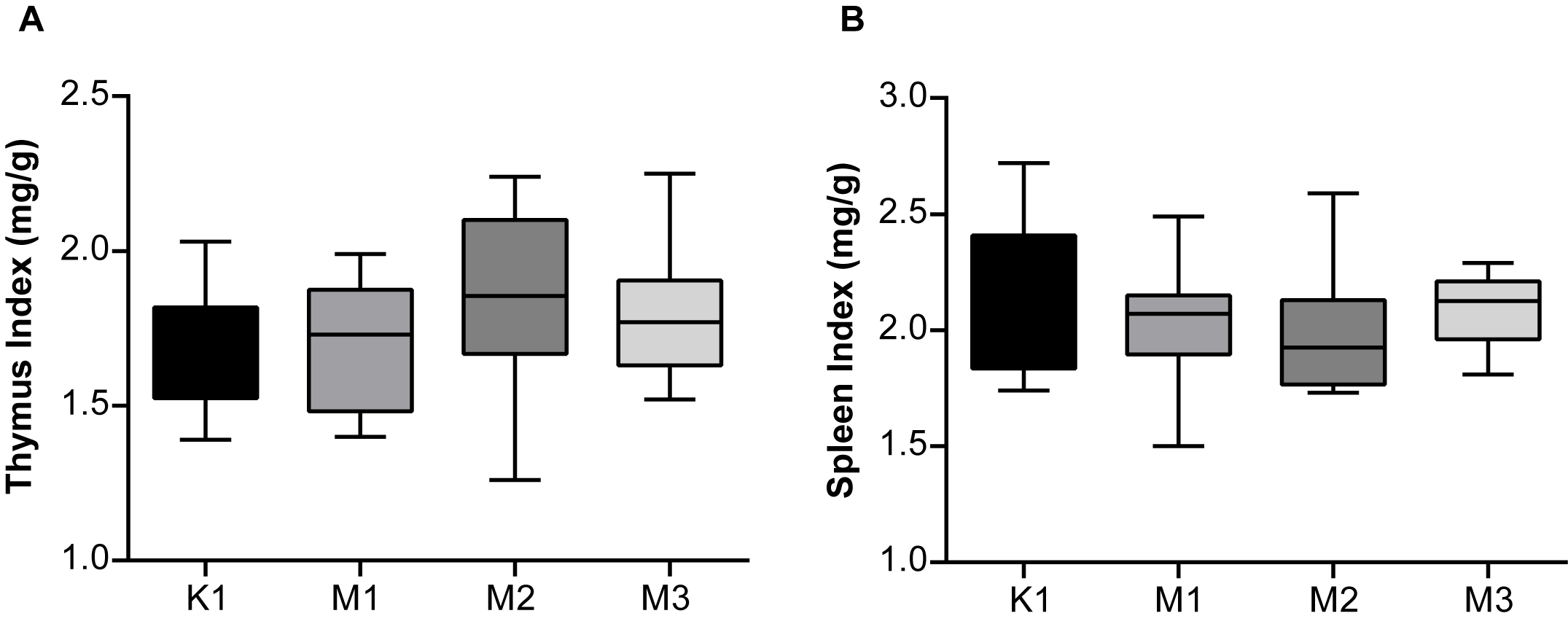

Supplement: Supplementary Figure 1 — Changes in thymus index (A) and spleen index (B) in rats in each group (n = 10). K1, normal control group; M1, 3-day model group; M2, 7-day model group; M3, 14-day model group. [file Image1.tif]

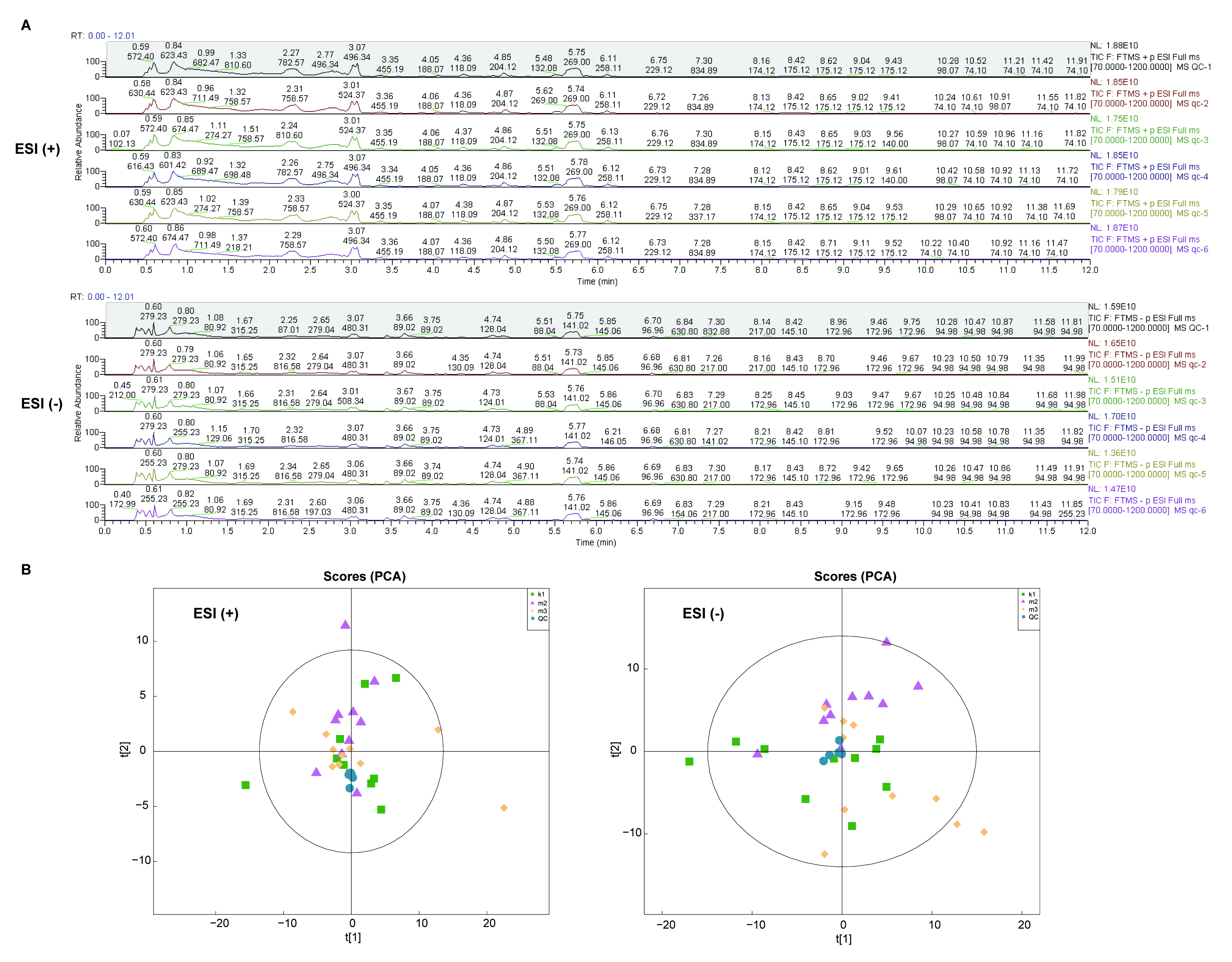

Supplement: Supplementary Figure 2 — Peak intensity chromatograms of serum samples in positive and negative modes. (A): Total ion chromatogram. (B): Metabolic profiles of the serum samples in the PCA plot. PCA, principal component analysis; ESI, electrospray ionization. [file Image2.tif]
